# CDYL promotes the chemoresistance of small cell lung cancer by regulating H3K27 trimethylation at the CDKN1C promoter

**DOI:** 10.7150/thno.33680

**Published:** 2019-07-09

**Authors:** Zhengang Qiu, Weiliang Zhu, Hui Meng, Lihua Tong, Xi Li, Peng Luo, Lilan Yi, Xiaoli Zhang, Linlang Guo, Ting Wei, Jian Zhang

**Affiliations:** 1 Department of Oncology, Zhujiang Hospital, Southern Medical University, Guangzhou 510282, Guangdong, China; 2 Department of Pathology, Zhujiang Hospital, Southern Medical University, Guangzhou 510282, Guangdong, China; 3 Department of Oncology, First Affiliated Hospital of Gannan Medical University, Ganzhou 341000, Jiangxi, China; 4 Department of Oncology, Nanhai Hospital Affiliated to Southern Medical University, Foshan 528200, Guangdong, China

**Keywords:** small cell lung cancer, chemoresistance, CDYL, CDKN1C, H3K27me3

## Abstract

**Rationale**: Chemoresistance frequently occurs in patients with small cell lung cancer (SCLC) and leads to a dismal prognosis. However, the mechanisms underlying this process remain largely unclear.

**Methods**: The effects of chromodomain Y-like (CDYL) on chemoresistance in SCLC were determined using Western blotting, immunohistochemistry, cell counting kit-8 assays, flow cytometry, and tumorigenicity experiments, and the underlying mechanisms were investigated using mRNA sequencing, chromatin immunoprecipitation-qPCR, electrophoretic mobility shift assays, co-immunoprecipitation, GST pull down assays, bisulfite sequencing PCR, ELISA, and bioinformatics analyses.

**Results**: CDYL is expressed at high levels in chemoresistant SCLC tissues from patients, and elevated CDYL levels correlate with an advanced clinical stage and a poor prognosis. Furthermore, CDYL expression is significantly upregulated in chemoresistant SCLC cells. Using gain- and loss-of-function methods, we show that CDYL promotes chemoresistance in SCLC in vitro and in vivo. Mechanistically, CDYL promotes SCLC chemoresistance by silencing its downstream mediator cyclin-dependent kinase inhibitor 1C (CDKN1C). Further mechanistic investigations showed that CDYL recruits the enhancer of zeste homolog 2 (EZH2) to regulate trimethylation of lysine 27 in histone 3 (H3K27me3) at the CDKN1C promoter region and promotes transcriptional silencing. Accordingly, the EZH2 inhibitor GSK126 de-represses CDKN1C and decreases CDYL-induced chemoresistance in SCLC.

**Principal conclusions**: Based on these results, the CDYL/EZH2/CDKN1C axis promotes chemoresistance in SCLC, and these markers represent promising therapeutic targets for overcoming chemoresistance in patients with SCLC.

## Introduction

Small cell lung cancer (SCLC) is a highly lethal disease that accounts for 13-15% of lung cancers 1, 2. Chemotherapy based on platinum and etoposide (EP doublet) is the current first-line treatment for affected patients 3. Frustratingly, the initial robust benefit of this treatment is frequently compromised by chemoresistance 4, 5, which is associated with a 5-year survival rate of less than 7% 6. Therefore, it is imperative to explore the precise molecular mechanisms of chemoresistance and to develop effective targeted therapies to diminish the SCLC chemoresistance.

CDYL (Chromodomain Y-like) is located on human 6p25.1, and the length of CDS is 1797 bp 7. This newly identified epigenetic regulator possesses an N-terminal chromodomain and a carboxy-terminal enoyl-coenzyme A hydratase-isomerase catalytic domain 7-9. It also acts as a transcriptional corepressor regulating the expression of its downstream genes, including RhoA, BDNF, SCN8A, and VGF 10-13. Recently, studies have shown that CDYL is involved in tumorigenesis. For example, Mulligan *et al.* found that CDYL knockdown increased the expression of proto-oncogene TrkC and induces oncogenic cellular transformation in human mammary epithelial cells in semisolid media 14. Studies also show that CDYL is required for silencing the E-cadherin gene, whose loss is an essential event in epithelial-mesenchymal transition and is crucial for invasion in malignant epithelial tumors 15. However, whether CDYL affects chemoresistance in tumors remains unknown.

Our previous cDNA microarray analysis revealed that the CDYL is differentially expressed between chemosensitive and chemoresistant SCLC cells (Figure S1A). In the following study, using patient tissue samples, cell lines and xenograft models, we reveal that high CDYL levels promote chemoresistance in SCLC. To further explore the mechanism of CDYL-regulated SCLC chemoresistance, we performed mRNA sequencing between CDYL-depleted SCLC cells and control cells and further identified cyclin-dependent kinase inhibitor 1C (CDKN1C, P57Kip2) as a targeted gene of CDYL in a series of experiments. Furthermore, we confirmed that CDKN1C repression is required for the CDYL-mediated SCLC chemoresistance. We next investigate the molecular basis of CDYL-induced CDKN1C silencing. As a member of cyclin-dependent kinase inhibitors (CDKIs) 16, CDKN1C expression can usually be regulated by H3K27me3 of the promoter 17, 18. A growing number of studies has also show that CDYL regulates the H3K27me3 of its downstream gene promoters by recruiting histone methyltransferase EZH2 10, 19. Thus, we boldly speculate that CDYL promotes SCLC chemoresistance by regulating H3K27me3 of the CDKN1C promoter via coordinating with EZH2, which was confirmed by the following assays.

Taken together, we identified CDYL as a novel chemoresistance-related gene, and the mechanism of which is that CDYL promotes chemoresistance by regulating H3K27me3 of the CDKN1C promoter under the coordination of EZH2. Additionally, we also found that EZH2 inhibition could decrease CDYL-induced SCLC chemoresistance.

## Methods and Materials

### Patients and tissue samples

A total of 82 SCLC patient tissues were available from Zhujiang Hospital (Guangzhou, China) during the period between January 2008 and January 2016. Patient samples were divided into 'chemosensitive' (partial or complete response) and 'chemoresistant' (progressive disease) groups based on the Response Evaluation Criteria in Solid Tumors (RECIST Edition 1.1). The agreement of every subject was obtained, and the experimental protocols complied with the standards of the Declaration of Helsinki.

### Cell lines and cell transfection

The human SCLC cell lines NCI-H69, NCI-H69AR and NCI-H446 were acquired from the American Type Culture Collection (ATCC, USA). Chemoresistant H446DDP cells were acquired by incubating H446 cells in progressively increasing doses of cisplatin (up to 5 μg/mL) over a period of 6 months. Cells were transiently transfected with siRNAs for CDKN1C (GenePharma, Shanghai, China) by using Lipofectamine 2000 and OPTI-MEM I (Invitrogen, USA) according to the manufacturer's instructions. For stable expression, lentiviral particles expressing shRNA for CDYL (shCDYL#1 and shCDYL#2), LV5-CDYL, and pcDNA3.1-CDKN1C (GenePharma, Shanghai, China) were transfected into SCLC cells. The sequences of shRNA and siRNA are listed in Table S1-2.

### RNA extraction and qRT-PCR

Total RNA was extracted from cell lines and tissues using TRIzol reagent (Invitrogen, USA), the RT Reagent Kit with gDNA Eraser (TaKaRa, Japan) and SYBR Premix ExTaq^TM^ (TaKaRa, Japan). The RNA concentrations were determined using a NanoDrop 2000 (Thermo). The qRT-PCR assays were performed in an ABI 7500 (Foster City, CA, USA) using SYBR Green (TaKaRa, Japan). The relative expression levels of target genes were normalized against the control, and fold changes were calculated through relative quantification (2^-ΔΔCt^). The sequences of qPCR primers (Sangon, Shanghai, China) are listed in Table S3.

### Western blot

Protein was extracted from cells using RIPA lysis buffer (Beyotime Biotechnology, Shanghai, China). Western blot antibody: CDYL (ab5188, Abcam), EZH2 (ab150433, Abcam), CDKN1C (ab75974, Abcam), and β-actin (ab8227, Abcam) were used, followed by the appropriate peroxidase-linked secondary goat anti-rabbit IgG antibody. The immune complexes were detected by chemiluminescence 20.

### Immunohistochemistry (IHC)

Tissue samples were deparaffinized and rehydrated. After treatment with endogenous peroxidase blocking solution, they were treated with specific antibodies against CDYL (ab5188, Abcam) and CDKN1C (ab75974, Abcam) overnight at 4°C. After they were washed with PBS, the samples were treated with horseradish peroxidase-conjugated anti-rabbit IgG and were then stained with diaminobenzidine. Expression levels were scored by multiplying the percentage of positive cells by the staining intensity. The percent positivity was scored as 0 if < 5% (negative), 1 if 5-30% (sporadic), 2 if 30-70% (focal) and 3 if 70% (diffuse) of the cells were stained; and staining intensity was scored as 0 for no staining, 1 for weak to moderate staining and 2 for strong staining. A score ≥ 2 was regarded as 'high', and a score < 2 was regarded as 'low' in immunohistochemical staining 21. Moreover, we used Image-pro Plus 6.0 Software (Media Cybernetics, Silver Spring, MD, USA) to conduct a semi-quantitative analysis of the staining intensity. We first selected the area of interest (AOI) in the stained area, measured the IOD (integrated optical density) in the area, and then divided the IOD by the area of the target region to obtain the average density of staining for the target protein (CDYL and CDKN1C).

### Cell counting kit-8 (CCK-8) assay

SCLC cells were cultured at 5 × 10^3^ cells per well in a 96-well plate with cytotoxic drugs for 24 h. Cytotoxic drugs (cisplatin and etoposide) were diluted to obtain different concentration gradients. Absorbance was detected at 450 nm after treatment with 10 μL of CCK-8 reagent (Dojindo, Kumamoto, Japan) for 4 h. The experiments were performed with five replicate wells per sample, and the assays were conducted in triplicate.

### Cell apoptosis and cell-cycle assay

For the cell apoptosis assay, SCLC cells were incubated with cytotoxic drugs (5 μg/ml DDP and 200 μg/ml VP-16 for 24 h) after they were harvested and washed. Cells were then resuspended with binding buffer containing propidium iodide (556463, BD Pharmingen, USA) and Annexin V-APC (640919, BioLegend, USA). For the cell-cycle assay, cells were collected and fixed with 70% ethanol at 4 °C for 24 h and then stained with propidium iodide. The results were analysed using flow cytometry and calculated as the means ± SD from of at least three independent experiments.

### Mouse xenograft experiment

The experiment was approved by the Institutional Guidelines and Use Committee for Animal Care of Guangdong province. Male nude mice (12-16 g, 4-6 weeks old) were purchased from Silaike Biotechnology Co. Ltd. (Hunan, China). Each group had five mice (n = 5). The mice received a subcutaneous injection in the back of SCLC cells (1 × 10^7^ cells/ 100 μL PBS). After that, the mice received intraperitoneal injections with etoposide (7 mg/kg body weight once every 2 days) and cisplatin (3 mg/kg body weight once every 7 days). The mice were sacrificed and stored on day 30. The sizes of the tumors were measured and recorded every 3 days by the following equation: V=1/2 (width ^2^ × length).

### cDNA microarray and mRNA sequencing

cDNA microarray assays were performed as previously described 22, and mRNA-sequencing assays were performed using a BGISEQ-500 platform (BGI Genomics, Wuhan, China).

### Chromatin immunoprecipitation quantitative PCR (ChIP-qPCR) assay

ChIP assays were conducted as previously described 23. CDYL (ab5188, Abcam), EZH2 (ab195409, Abcam) and H3K27me3 antibodies (9733, CST) were used. Immunoprecipitated DNA extracted from SCLC cells was analyzed by qPCR. The primers are listed in Table S4.

### Electrophoretic mobility shift assay (EMSA)

EMSAs were conducted as described in a previous study 24. A commercially available recombinant CDYL protein (H00009425-P01, Abnova, Taiwan) and the nuclear proteins of SCLC cells were used in the assays. Sixty femtomoles of labelled probes containing CDKN1C sequences were hybridized with 5 μL of recombinant CDYL protein and nuclear proteins for 20 min at 20°C. The mixtures were then subjected to electrophoretic separation on a 6% polyacrylamide gel at 4°C, transferred to a nylon membrane, and then visualized using chemiluminescence. The probe sequences are listed in Table S5.

### Coimmunoprecipitation (Co-IP)

Flag-tagged pGEX-4T-1-CDYL and Myc-tagged pET-28a (+)-EZH2 recombinant plasmids were transformed into H69AR cells according to the manufacturer's protocol 25. Cells were collected after 48 h of transfection and lysed with RIPA lysis buffer. Lysates were mixed with the relevant antibodies overnight at 4°C. Antibodies/lysates were captured with 100 µl of Protein A/G beads, extensively washed with HEGNDT buffer, and then washed once in HEGNDT buffer without Triton X-100. The eluted bound proteins were detected by Western blot using Flag and Myc antibodies (CWBio, Beijing, China).

### GST pull down assay

CDYL cDNA was isolated by RT-PCR and cloned into the BamHI and XhoI sites of the GST-tagged pGEX-4T-1 vector 160 by using T4 DNA Ligase (Thermo Scientific, USA). EZH2 cDNA was cloned into the BamHI and XhoI sites of a GST (TransGen, Beijing, China)-tagged pET-28a (+) vector. The pGEX-4T-1-CDYL and pET-28a (+)-EZH2 recombinant plasmids were transformed into Escherichia coli BL21 (DE3) cells separately. PCR identification, double enzyme digestion, and sequencing were used to screen and identify highly expressing positive clones that synthesized GST-CDYL or Myc-EZH2 recombinant proteins. The purified GST-CDYL fusion proteins were attached to Glutathione Sepharose (GE Healthcare, USA) and were then incubated with purified Myc-EZH2 protein overnight at 4°C. The eluted bound proteins were detected by Western blot using GST (TransGen, Beijing, China) and Myc antibodies (CWBio, Beijing, China).

### SCLC cell line datasets

A total of 51 human SCLC cell lines as reported in the Genomics of Drug Sensitivity in Cancer (GDSC, Sanger Institute, United Kingdom) databases was investigated 26. The CDKN1C expression profiles and the corresponding IC50 values to cis-platinum from the cell lines were analyzed. An IC50 ≥ 10 μM was regarded as 'chemoresistant', and an IC50 < 10 μM is regarded as 'chemosensitive' according to the standards of the GDSC.

### Bisulfite sequencing PCR (BSP)

BSP was conducted as previously described 27. DNA from SCLC cells was isolated using the Genomic DNA Purification Kit (A1125, Promega wizard) and bisulfate modified with the EpiTect Bisulfite Kit (59104, QIAGEN) and was then used for BSP analysis. The primer sequences for BSP are listed in Table S6.

### Enzyme-linked immunosorbent assay (ELISA)

For the assessment of DNA methyltransferase (DNMT) activity, the nuclear proteins were extracted from SCLC cells using a nuclear extraction kit and quantified using a BCA Protein Assay Kit (Beyotime Biotechnology, Shanghai, China). Ten micrograms of nuclear extracts were used to measure DNMT activity according to the instructions provided with the ELISA kit (EpiQuik™ DNMT Activity/Inhibition Assay Ultra Kit, USA). The absorbance was measured at 450 nm using a microplate reader (Bio-Tek, Germany).

### Statistical analysis

The data were analyzed by SPSS 20.0 and GraphPad Prism 5.0 and are represented as the means ± SD. Comparisons of two groups were analyzed by Student's t-tests, comparisons of more than two samples were analyzed by ANOVA, and least-significant difference (LSD) tests were used to estimate multiple comparisons. *P* < 0.05 was considered statistically significant.

## Results

### CDYL is upregulated in chemoresistant SCLC tissues and correlates with the clinical stage and prognosis

**Figure 1:**
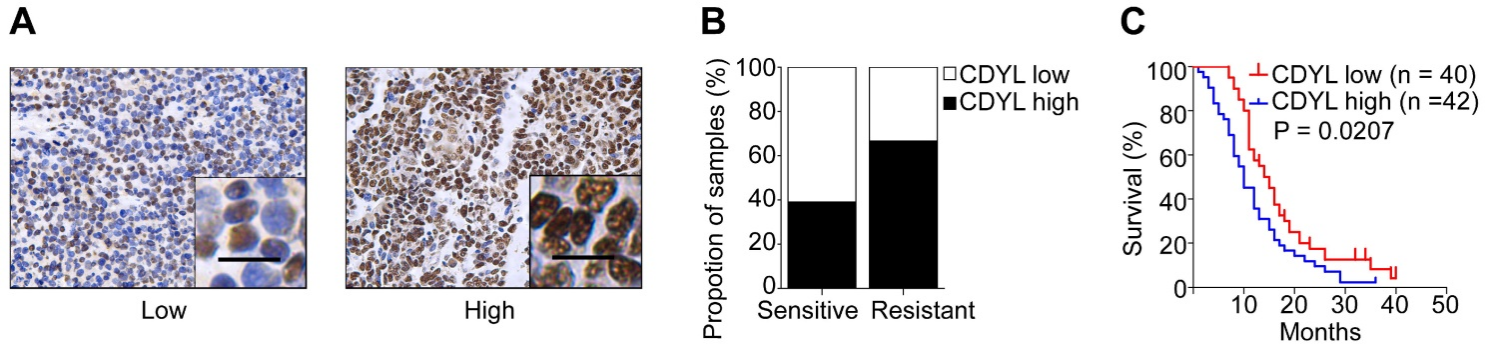
** CDYL levels and the clinical effect of CDYL on SCLC.** (A) Representative samples showing high and low intensity CDYL staining in 82 SCLC tissues. (B) Percentages of CDYL-high and CDYL-low samples among 45 chemosensitive and 37 chemoresistant SCLC tissues. Scale bar, 50 μm. Clinical data are presented in Table 1. (C) Kaplan-Meier analysis of the overall survival of 82 patients stratified by CDYL levels. n, number of patients, *P* = 0.0207.

In our previous study, we used a cDNA microarray to screen differentially expressed genes between drug-resistant and drug-sensitive cell lines 22. CDYL was expressed at high levels in chemoresistant SCLC cells (Figure S1A). To further evaluate the clinical importance of CDYL in SCLC, we analysed the CDYL levels in samples from 82 patients with SCLC using IHC. A greater percentage of samples from chemoresistant patients showed high CDYL expression (66.7%) than samples from chemosensitive patients (39.1%) (Figure 1A-B). Moreover, the Kaplan-Meier analysis revealed a correlation between high CDYL levels and poor overall survival (*P* = 0.0207) (Figure 1C). Finally, as indicated in Table 1, higher levels of the CDYL protein were observed in patients with extensive-stage SCLC than in patients with limited-stage SCLC (*P* = 0.0257) using IHC. The multivariate analysis showed that CDYL was an independent prognostic factor (*P* = 0.011, Table 2). Collectively, high CDYL levels correlate with a worse response to chemotherapy, poor survival, and more advanced tumour stages in patients with SCLC.

### CDYL promotes chemoresistance in SCLC in vitro and in vivo

**Figure 2:**
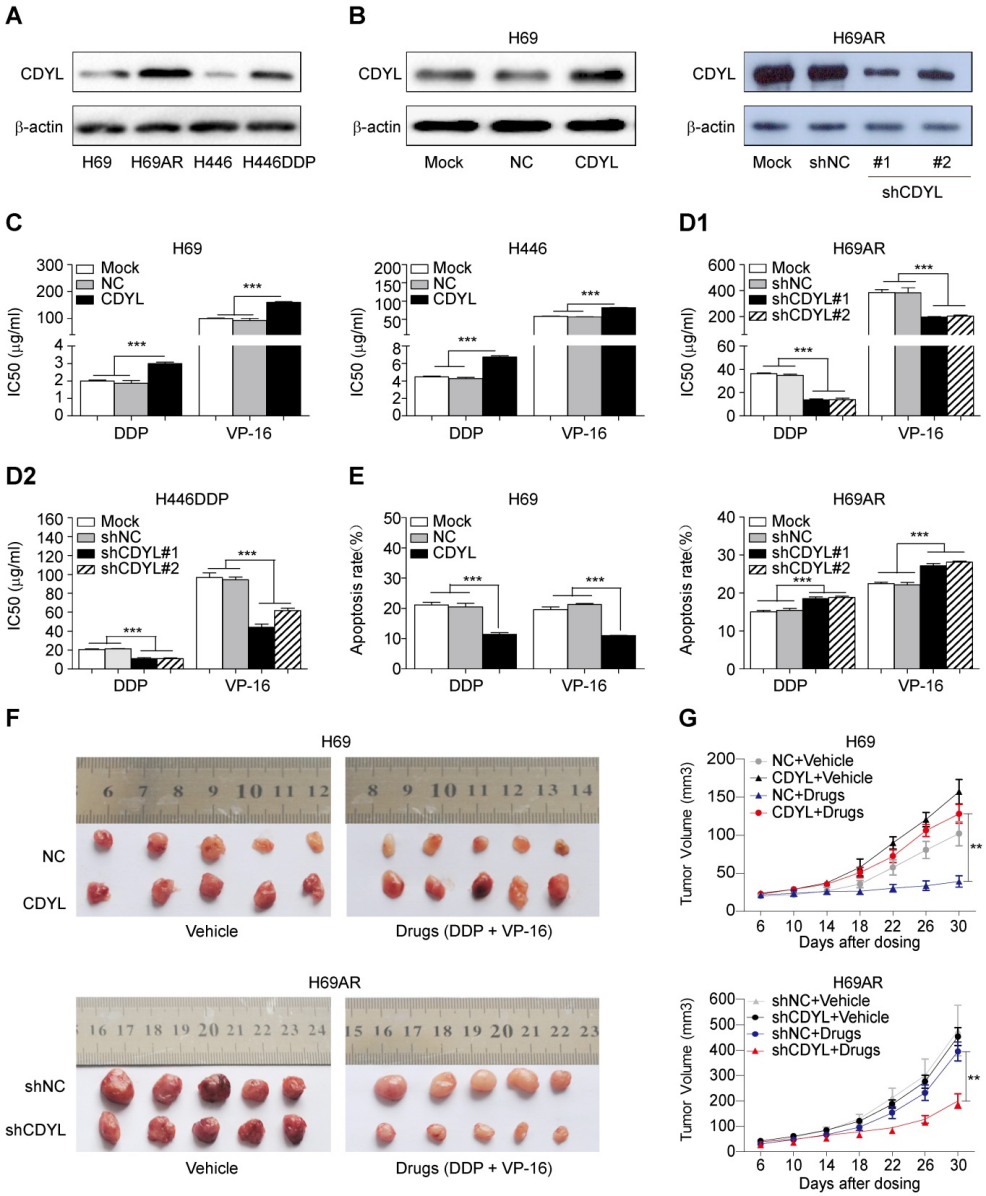
** Effect of CDYL on chemoresistance in SCLC in vitro and in vivo.** (A) Western blot showing CDYL levels in pairs of sensitive and resistant SCLC cell lines. (B) Western blot showing CDYL levels in H69 cells transfected with a LV5-CDYL lentivirus (left panel), H69AR cells transfected with shRNA-CDYL (right panel) and the corresponding control vectors. (C, D1 and D2) Comparison of IC50 values for cytotoxic agents [(DDP: cisplatin, 5 μg/ml; VP-16: etoposide, 200 μg/ml) for 24 h] in the SCLC cells shown in (B). (E) Summary of the cumulative data showing the percentage of apoptotic SCLC cells following 24 h of exposure to cytotoxic agents shown in (B). (F) Xenograft growth in nude mice injected with the cells shown in (B) and treatment with or without cytotoxic drugs (n = 5 mice per group). (G) Growth curve for tumour volumes in the mice shown in (F). ** *P* < 0.01.

We utilized two paired sensitive-resistant cell lines, H69-H69AR and H446-H446DDP, as models to assess the role of CDYL in the chemoresistance of SCLC in vitro 25. Both RT-qPCR and Western blots revealed significantly higher CDYL levels in chemoresistant cells than in chemosensitive cells (Figures S1B and 2A). Using gain- and loss-of-function methods (Figures 2B and S1C), we found that CDYL overexpression in chemosensitive cells markedly increased IC50 values (Figure 2C) and reduced apoptosis (Figures 2E, S1D-E, left panels) following exposure to cytotoxic drugs. In contrast, CDYL silencing in chemoresistant cells decreased IC50 values (Figure 2D1-D2) and increased cell apoptosis (Figures 2E, S1D-E, right panels). Based on these data, CDYL promotes chemoresistance in SCLC in vitro.

We next established mouse xenograft models using SCLC cells with altered CDYL expression to determine whether CDYL regulates chemoresistance in vivo. The tumour-bearing mice were intraperitoneally injected with chemotherapeutic drugs (DDP + VP-16). Significantly larger tumour volumes and significantly higher growth rates were observed in the CDYL overexpression group than in the corresponding control group after chemotherapy treatment (Figure 2F-G, top panels). In contrast, CDYL knockdown significantly reduced the tumour volumes and growth rates (Figure 2F-G, bottom panels). Thus, CDYL confers chemoresistance in SCLC in vivo.

### CDYL directly targets CDKN1C in SCLC

**Figure 3:**
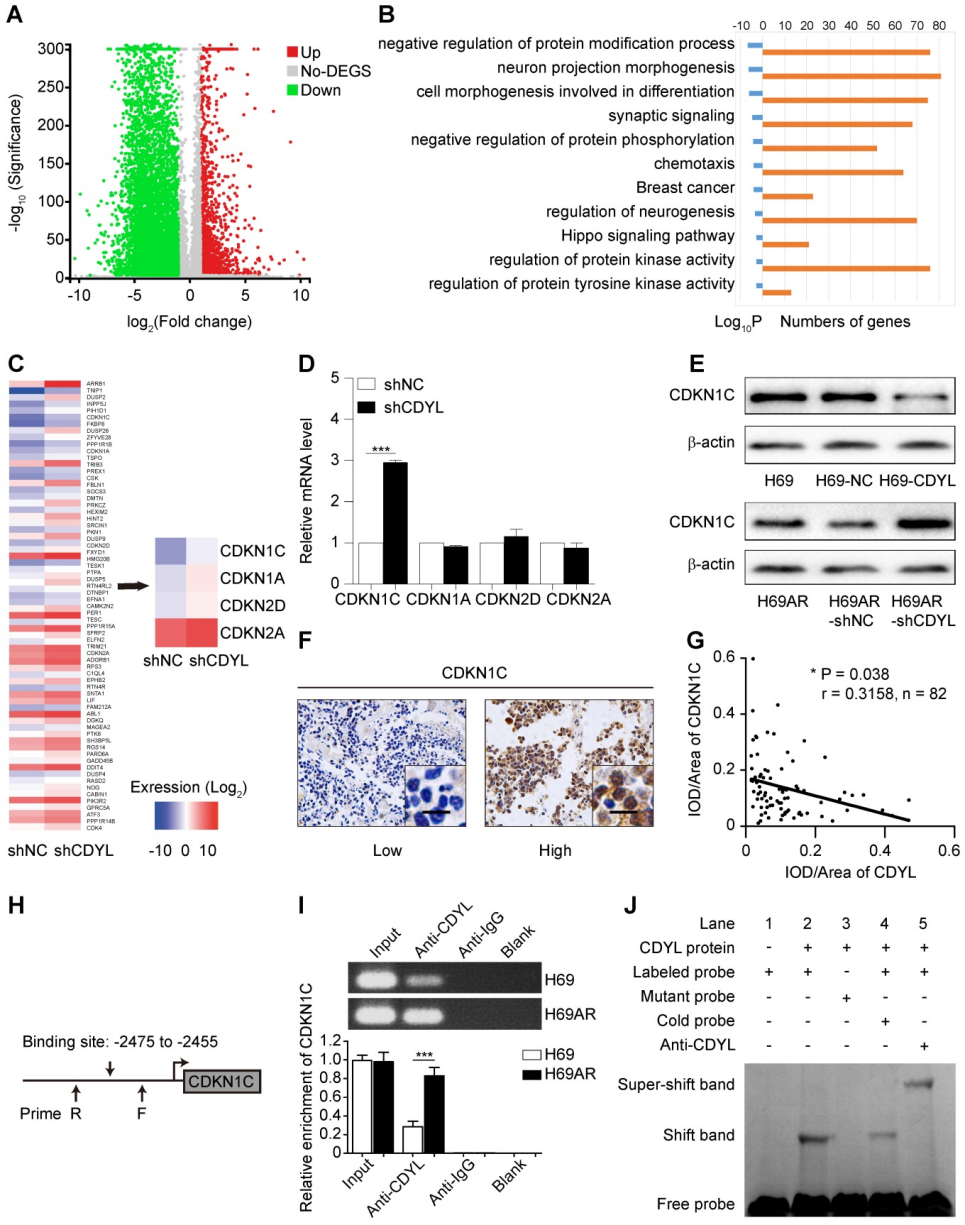
** CDYL directly targets CDKN1C in SCLC.** (A) Volcano plot showing the differentially expressed genes between H69AR-shCDYL cells and H69AR-shNC cells. Significantly differentially expressed genes were determined based on a |fold change| ≥ 2. (B) -Log2 transformations of the *P*-values of the top 10 significantly upregulated pathways. (C) Heat maps showing all 68 differentially expressed genes (left panel) and the 4 differentially expressed CDKIs (right panel) in a pathway that negatively regulates protein modifications between H69AR-shCDYL cells and control cells (fold enrichment > 2). (D) RT-qPCR analysis of CDKN1C, CDKN1A, CDKN2A, and CDKN2D expression in H69AR-shCDYL cells and control cells. (E) Western blot showing CDKN1C levels in SCLC cells with different CDYL levels. (F) Representative samples showing high and low intensity CDKN1C staining in 82 SCLC tissues. (G) Spearman's correlation analysis of the IHC staining for CDYL and CDKN1C (r: correlation coefficient; *P* = 0.038). (H) Predicted CDYL binding site and the qPCR primer location in the CDKN1C promoter region. (I) CDYL ChIP-qPCR assessing CDYL enrichment at the CDKN1C promoter. (J) CDYL EMSA assessing the binding of recombinant CDYL to the CDKN1C promoter.

We first performed mRNA sequencing to screen differentially expressed genes between CDYL-depleted SCLC cells and control cells and investigate the molecular mechanism by which CDYL regulates chemoresistance in SCLC. A total of 7924 differently expressed genes were identified (|fold change| ≥ 2.00 and FDR ≤ 0.001) (Figure 3A). Because CDYL functions as a transcriptional corepressor 28, we speculated that the significantly upregulated genes were more likely to be direct target genes of CDYL. Thus, we focused on the 1609 upregulated genes in CDYL-depleted SCLC cells and then conducted a gene set enrichment analysis (Figure 3B). We selected the most significantly enriched pathway, negatively regulated protein modification, for further study (Figure 3B). Four cyclin-dependent kinase inhibitor (CDKI) genes (CDKN1C, CDKN1A, CDKN2D, and CDKN2A) are involved in this pathway (Figure 3C). Because our previous study revealed the differential expression of CDKI genes between chemoresistant H69AR SCLC cells and chemosensitive H69 SCLC cells 22, we then focused on the four candidate CDKI genes. Both RT-qPCR and Western blots showed significant increases in CDKN1C levels, but not CDKN1A, CDKN2D, and CDKN2A levels, in CDYL-deficient SCLC cells (*** *P* < 0.001) (Figure 3D-E). This finding was also confirmed by a subsequent IHC analysis of xenografts (Fig S2D), Furthermore, a correlation analysis of IHC staining in samples from 82 patients with SCLC revealed a negative correlation between CDYL expression and CDKN1C expression (Figure 3F-G).

We first predicted the CDYL binding site (-2475 to -2455 bp) in the CDKN1C promoter region based on the JASPAR CORE database and previous studies 13, 19 to further explore the interaction between CDYL and CDKN1C (Figure 3H). We next performed CDYL ChIP-qPCR targeting the CDKN1C gene in SCLC cells. CDYL bound to CDKN1C, and H69AR cells exhibited markedly increased CDYL enrichment at the CDKN1C promoter (Figure 3I). We performed a CDYL EMSA to further confirm the binding of CDYL to the CDKN1C promoter in vitro and determined that CDYL protein directly bound to the CDKN1C promoter in vitro (Figures 3J and S2E). Taken together, these data confirm that CDYL directly targets CDKN1C and negatively regulates its expression.

### CDKN1C contributes to CDYL-mediated chemoresistance in SCLC

**Figure 4:**
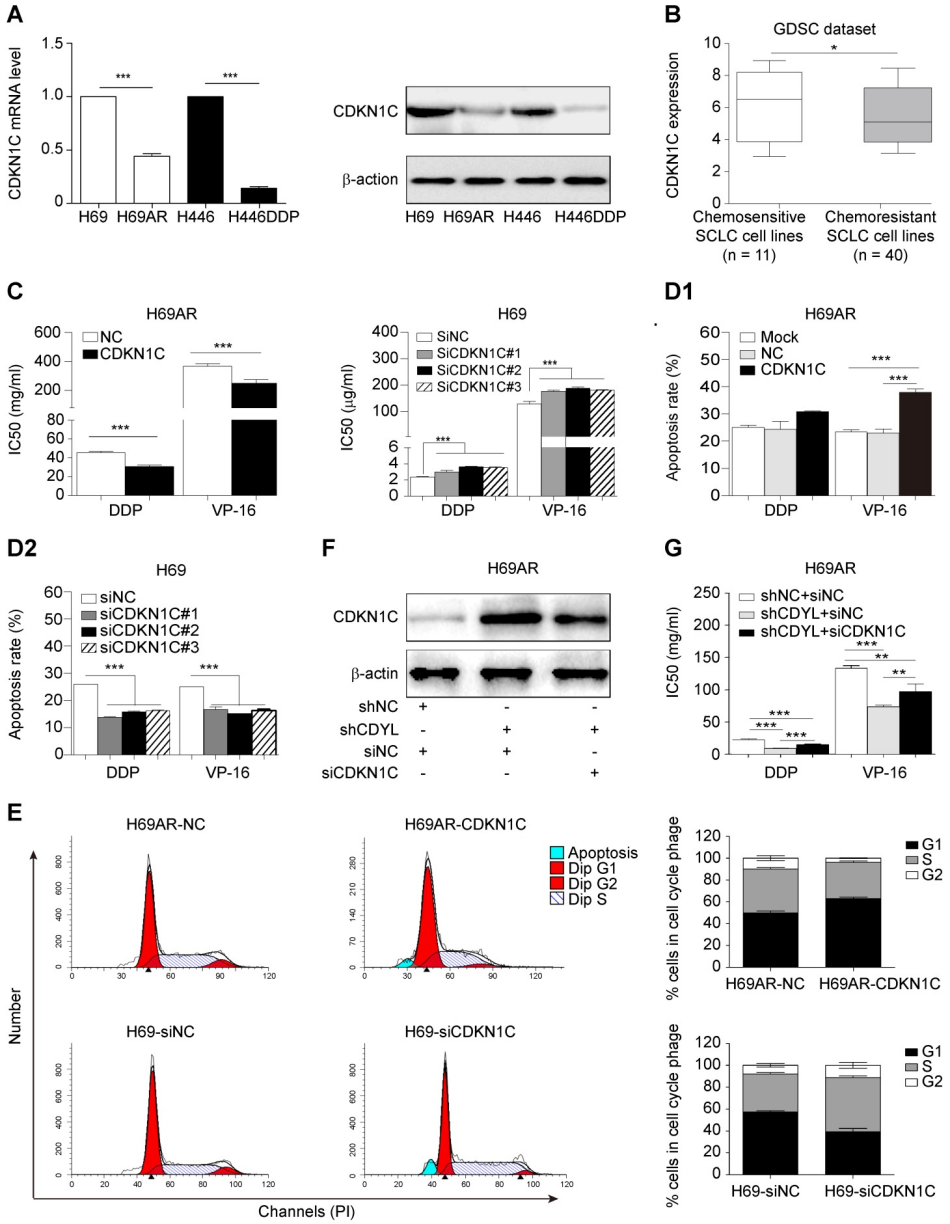
** CDKN1C repression mediates CDYL-induced chemoresistance in SCLC.** (A) RT-qPCR and Western blot analyses of CDKN1C levels in paired H69-H69AR and H446-H446DDP SCLC cells. (B) Comparison of CDKN1C expression in chemosensitive SCLC cell lines (n = 11) and chemoresistant SCLC cell lines (n = 40) (*P* = 0.0476) from the GDSC datasets. (C) Comparison of IC50 values following exposure of H69AR-CDKN1C (left panel) or H69-siCDKN1C (right panel) cells and corresponding controls to cytotoxic agents. (D) A summary of the cumulative data showing the percentage of apoptotic H69AR-CDKN1C (D1) and H69-siCDKN1C (D2) SCLC cells and corresponding controls. (E) Cell cycle progression was determined in CDKN1C-overexpressing and CDKN1C knockdown SCLC cells after exposure to chemotherapeutic drugs using flow cytometry. (F) Western blot showing CDKN1C levels in H69AR cells transfected with shCDYL, siRNA-CDKN1C and the corresponding control vectors. (G) CCK8 analysis of the IC50 values for cytotoxic agents in the cells shown in (E). ** *P* < 0.01 and *** *P* < 0.001.

As CDKN1C is the direct target gene of CDYL in SCLC cells, we sought to investigate whether CDKN1C mediates CDYL-induced SCLC chemoresistance. Significantly higher levels of the CDKN1C mRNA and protein were observed in chemosensitive cells than in the corresponding chemoresistant cells (Figure 4A). Interestingly, CDKN1C was expressed at higher levels in 11 chemosensitive SCLC cell lines than in 40 chemoresistant SCLC cell lines in the GDSC datasets (*, *P* = 0.0476) (Figure 4B). Using gain- and loss-of-function methods (Figure S2A-B), we found that CDKN1C overexpression in chemoresistant cells decreased IC50 values (Figs 4C, S2C, left panels) and increased cell apoptosis (Figure 4D1). In contrast, CDKN1C knockdown in chemosensitive cells led to markedly increased IC50 values (Figures 4C, S2C, right panels) and reduced apoptosis (Figure 4D2) after exposure to cytotoxic drugs, indicating that CDKN1C negatively regulates SCLC chemoresistance. Because CDKN1C is a cell cycle-related gene, we analysed the effects of CDKN1C and CDYL on the cell cycle distribution. CDKN1C overexpression and CDYL knockdown significantly increased the G1 arrest and inhibited cell cycle progression (Figure 4E, top panels and Figure S1F, right panel). In contrast, CDKN1C knockdown and CDYL overexpression significantly decreased the G1 arrest and promoted cell cycle progression (Figure 4E, bottom panels and Figure S1F, left panel). Moreover, rescue experiments showed that the decreased IC50 for cytotoxic agents observed in CDYL-deficient H69AR cells was rescued by siRNA-mediated knockdown of CDKN1C (Figure 4F-G), indicating that CDYL mediated the chemoresistance of SCLC cells through its downstream mediator CDKN1C. These results confirmed that CDKN1C contributes to CDYL-mediated chemoresistance in SCLC.

### CDYL regulates H3K27 trimethylation at the CDKN1C promoter in coordination with the histone methyltransferase EZH2

**Figure 5:**
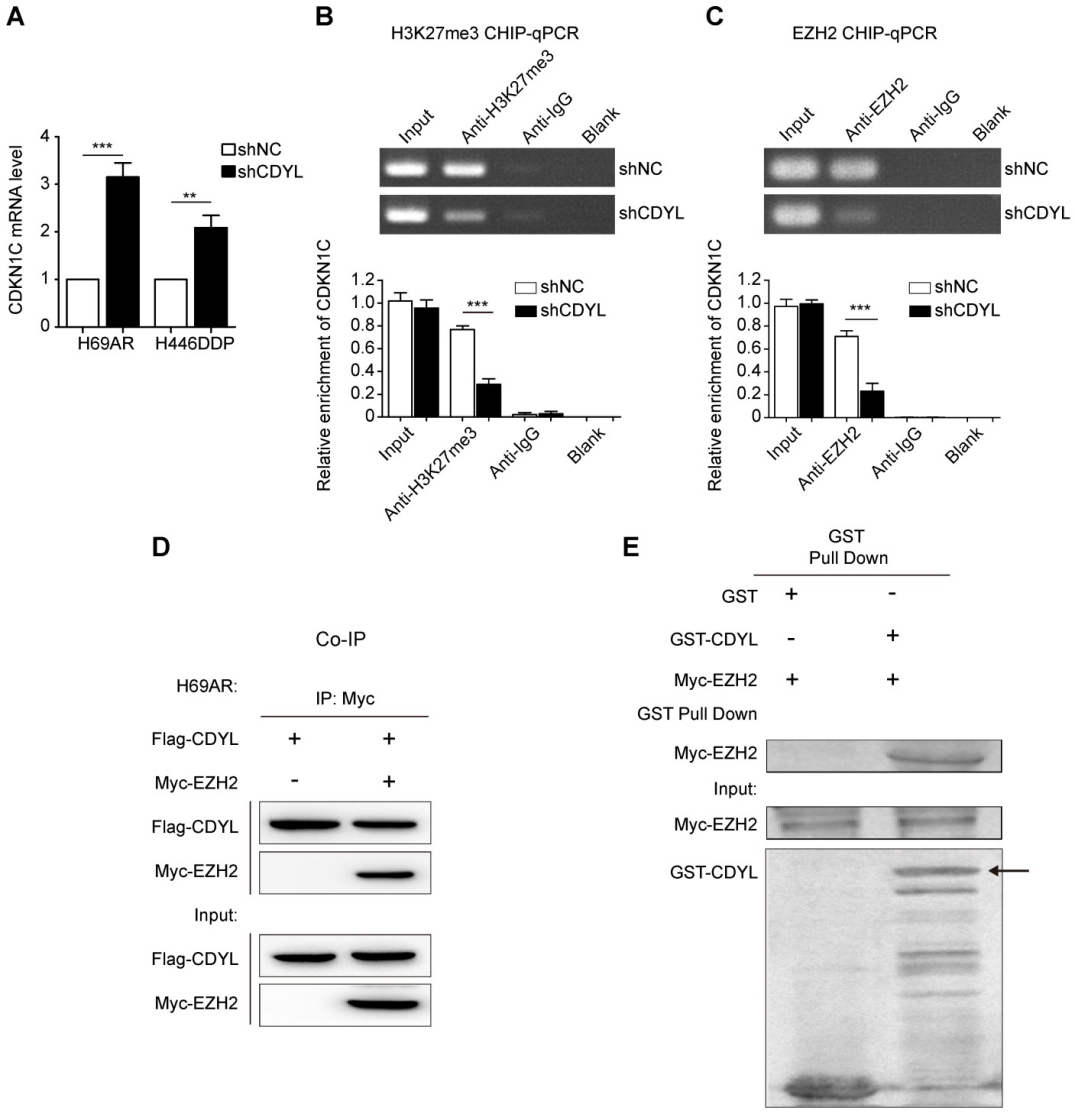
**The EZH2-mediated H3K27me3 pathway regulates CDYL-induced CDKN1C repression**. (A) RT-qPCR analysis of CDKN1C expression in shCDYL cells and control cells. (B) H3K27me3 ChIP-qPCR assessing H3K27me3 enrichment at the CDKN1C promoter, *** *P* < 0.001. (C) EZH2 ChIP-qPCR assessing the binding of EZH2 to the CDKN1C promoter; CDYL knockdown reduced the binding of EZH2 to the CDKN1C promoter and increased CDKN1C expression. *** *P* < 0.001. (D) Co-immunoprecipitation analysis of CDYL and EZH2 in H69AR SCLC cells. (E) GST pull down assay testing the interaction between the CDYL and EZH2 proteins.

We next sought to determine the molecular mechanisms by which CDYL silences CDKN1C expression. CDYL regulates the H3K27me3 level at the promoters of downstream genes by recruiting histone methyltransferase EZH2 10, 19. Thus, CDYL-mediated CDKN1C repression also likely occurs through the EZH2-H3K27me3 pathway. We performed RT-qPCR experiments and observed significantly increased levels of the CDKN1C mRNA in CDYL knockdown SCLC cells (Figure 5A). We next performed ChIP-qPCR using antibodies against H3K27me3 and EZH2. CDYL silencing markedly reduced the level of H3K27me3 at the CDKN1C promoter (Figure 5B). Moreover, the EZH2 ChIP-qPCR results showed that EZH2 bound to the CDKN1C promoter, and CDYL knockdown reduced CDKN1C repression (Figure 5C), indicating that CDKN1C was transcriptionally silenced by EZH2-mediated H3K27me3 at the CDKN1C promoter.

We performed co-immunoprecipitations to further evaluate the relationship between CDYL and EZH2, and confirmed the interaction between CDYL and EZH2 in H69AR SCLC cells (Figure 5D). Furthermore, the two proteins directly interacted in a GST pull down experiment (Fig 5E), suggesting that CDYL interacted with EZH2 in vitro. Additionally, we performed a BSP analysis to detect differences in the DNA methylation states of the CDKN1C promoter region between CDYL knockdown cells and control cells. CDYL knockdown did not significantly affect the DNA methylation level of the CDKN1C promoter and the total DNMT activity (Figure S3A-B).

Collectively, CDYL recruits EZH2 to regulate H3K27me3 at the CDKN1C promoter.

### EZH2 inhibition decreases CDYL-induced chemoresistance

**Figure 6:**
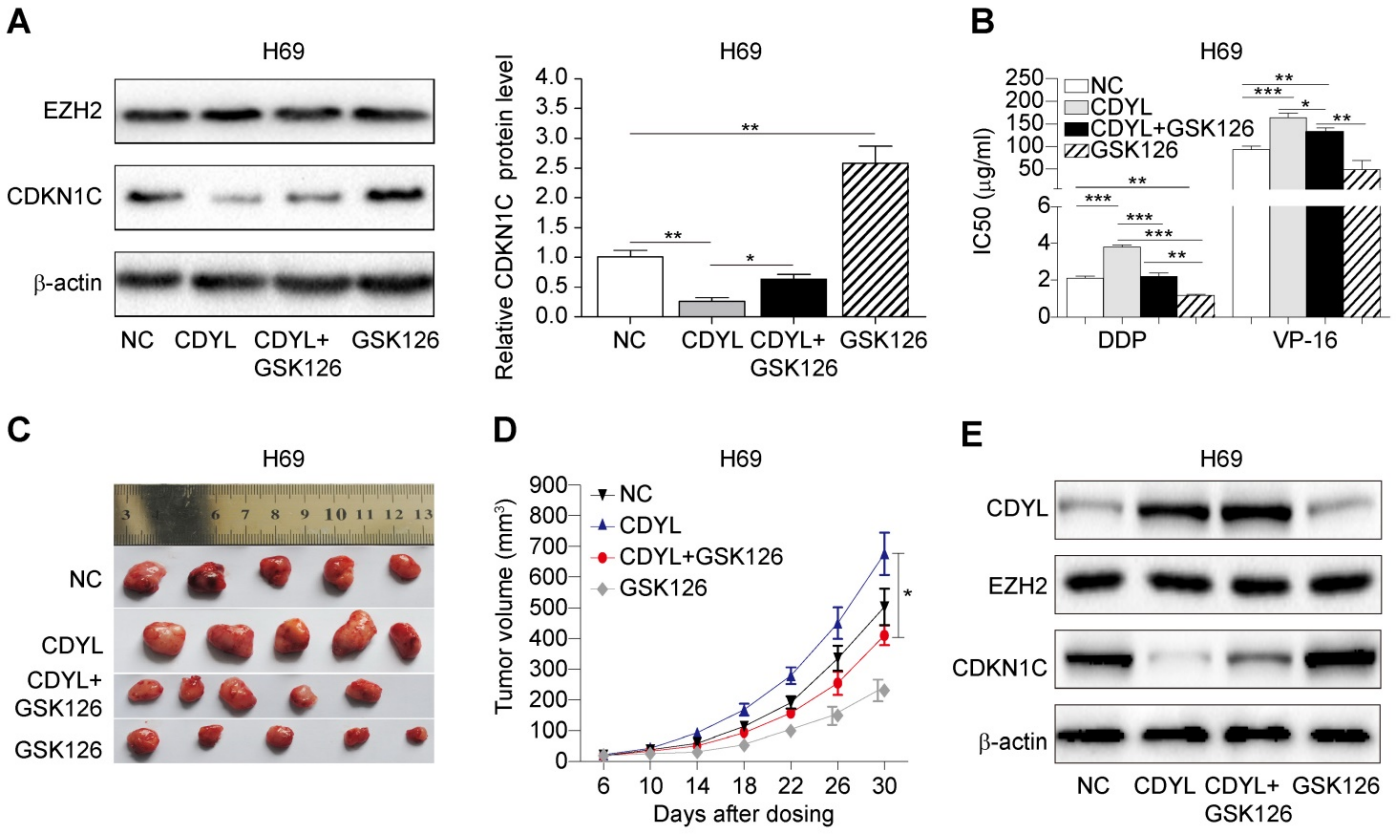
** Effect of the EZH2 inhibitor on CDYL-induced chemoresistance.** (A) Western blots showing EZH2 and CDKN1C levels in H69 cells and CDYL-overexpressing H69 cells treated with or without GSK126 (left panel) and quantification of CDKN1C levels (right panel). (B) CCK8 analysis of IC50 values in the H69 cells shown in (A). * *P* < 0.05, ** *P* < 0.01, and *** *P* < 0.001. (C) Effects of chemotherapy with or without GSK126 on tumour growth in mice injected with H69 cells and CDYL-overexpressing H69 cells (n = 5 animals per group). (D) Tumour growth curve for the mice shown in (D). * *P* < 0.05. (E) Western blots showing CDYL, EZH2 and CDKN1C levels in xenograft tumours.

Based on the results presented above, CDYL regulates SCLC chemoresistance by coordinating with EZH2; therefore, we hypothesized that treatment with an EZH2 inhibitor might prevent CDYL-induced chemoresistance. We used the selective EZH2 inhibitor GSK126 in subsequent experiments. We performed Western blots of lysates from treated H69 cells and found that GSK126 significantly increased CDKN1C levels in CDYL-overexpressing H69 cells (Figure 6A). Moreover, the CCK8 results showed that the increased IC50 values in CDYL-overexpressing cells were rescued by GSK126 (Figure 6B). Similarly, xenograft experiments also showed that the increased xenograft growth and tumour volumes observed after CDYL overexpression in H69 cells were significantly inhibited by the combination of GSK126 and chemotherapy (Figure 6C-D). We also performed Western blot analyses to detect the levels of CDYL, EZH2, and CDKN1C in xenograft tumours, and GSK126 significantly increased CDKN1C levels in CDYL-overexpressing H69 cells, but did not significantly change CDYL levels (Figure 6E). Therefore, EZH2 inhibition decreases CDYL-induced chemoresistance in vitro and in vivo.

## Discussion

CDYL has been primarily identified as a key regulator of mammalian spermatogenesis and nervous system development 9, 10, 29. Recently, CDYL has also been reported to modulate tumour invasion and oncogenic cellular transformation 14, 15. However, researchers have not determined whether CDYL regulates chemoresistance. Our cDNA microarray expression profiles revealed the differential expression of CDYL between chemoresistant and chemosensitive SCLC cells. Then, CDYL was expressed at higher levels in the two chemoresistant SCLC cells than in parental chemosensitive SCLC cells. Down- or up regulation of CDYL increased or decreased SCLC chemoresistance, respectively, in vitro and in vivo. In addition, samples from patients with chemoresistant SCLC showed higher expression of CDYL than samples from patients with chemosensitive SCLC. CDYL has been previously described to function as a tumour suppressor in several types of cancer 14. However, in the present study, high CDYL levels correlated with an advanced clinical stages and poor prognosis, suggesting that CDYL may function as an oncogene in SCLC. Together, our results confirm that CDYL contributes to chemoresistance in SCLC and represents a new marker that influences disease progression and the prognosis of patients with SCLC.

The mRNA sequencing results preliminarily revealed that CDKN1C was a candidate targeted gene of CDYL. Following overexpression or silencing of CDYL, CDKN1C expression was decreased or increased, respectively. Furthermore, using CDYL ChIP-qPCR and EMSAs, we confirmed that CDKN1C was a direct target of CDYL. According to previous studies, CDKN1C mainly functions as an important regulator of cell proliferation and differentiation 30-32. In the present study, we confirmed that CDKN1C repression conferred SCLC chemoresistance. Finally, we preformed rescue experiments and found that CDKN1C was an essential mediator of CDYL-mediated chemoresistance in SCLC. Taken together, we determined that the CDYL-CDKN1C axis promotes chemoresistance in SCLC.

We further explored how CDYL regulates CDKN1C expression. CDYL regulates the chromatin substrate by reading the repressive mark H3K27me3 19, 33. Based on accumulating evidence, H3K27me3 is also one of the most common modifications promoting CDKN1C inactivation 34, 35. For instance, CDKN1C is targeted by H3K27me3 in breast cancer cells 36. Therefore, we speculated that CDYL likely induced CDKN1C silencing by regulating the H3K27me3 modification. As expected, a subsequent H3K27me3 ChIP-qPCR showed that the H3K27me3 enrichment at the CDKN1C promoter region was dramatically decreased in CDYL-depleted cells compared to control cells. Furthermore, our EZH2 ChIP-qPCR results revealed the binding of EZH2 to the CDKN1C promoter region, and CDYL knockdown reduced CDKN1C repression. Thus, the EZH2-H3K27me3 pathway participates in CDYL-mediated CDKN1C repression. We performed a protein interaction analysis to further examine the relationship between CDYL and EZH2, and confirmed that CDYL directly interacted with EZH2. Based on our results, CDYL recruits EZH2 to regulate H3K27me3 levels at the CDKN1C gene promoter, resulting in a repressive state that inhibits CDKN1C expression.

In the present study, CDYL promoted chemoresistance in SCLC through EZH2. We aimed to identify an existing inhibitor that has been proved to be effective at reducing CDYL-mediated chemoresistance in SCLC in clinical studies, and the EZH2 inhibitor GSK126 ultimately met the requirements. GSK126 significantly increased CDKN1C levels and reduced chemoresistance in CDYL-overexpressing H69 cells. Our data might have potential clinical implications for patients with refractory SCLC presenting high CDYL expression who may benefit from combination chemotherapy with an EZH2 inhibitor. Taken together, the CDYL/EZH2/CDKN1C axis promotes chemoresistance in SCLC, and these markers represent promising therapeutic targets for overcoming chemoresistance in patients with SCLC.

## Conclusions

In summary, CDYL promotes chemoresistance in SCLC by increasing H3K27 trimethylation at the CDKN1C promoter via EZH2. Our findings provide potential therapeutic targets for improving the efficacy of chemotherapy in patients with refractory SCLC presenting high CDYL expression.

## Supplementary Material

Supplementary figures and tables.

## Figures and Tables

**Table 1:** CDYL expression in 82 patients with SCLC and the associations with clinicopathological factors

Clinicopathological features	N	Expression of CDYL		
		+	-	*P*
Gender				0.641
Male	67	33	34	
Female	15	9	6	
Age				0.808
<60	45	22	23	
≥60	37	20	17	
Clinical Stages				0.0257
Limited disease	44	17	27	
Extensive disease	38	25	13	

-, low expression; +, high expression of CDYL. *P*-values were calculated using Pearson's χ^2^-test. *P* < 0.05 was considered statistically significant.

**Table 2:** Univariate and multivariate analyses of potential prognostic factors associated with the overall survival of patients with SCLC

	Univariate analysis				Multivariate analysis		
Variables	Hazard ratio	95%CI	*P* value		Hazard ratio	95%CI	*P* value
Gender	0.962	0.514-1.802	0.904		0.854	0.444-1.641	0.635
Age	0.879	0.562-1.376	0.573		0.778	0.489-1.237	0.288
Clinical stages	1.935	1.224-3.058	0.005		1.951	1.164-3.272	0.011
Chemoresistance	1.856	1.173-2.936	0.008		1.792	1.091-2.945	0.021
CDYL expression	2.491	1.558-3.982	0.000		1.880	1.139-3.105	0.014

CI, confidence interval. *P*-values were calculated using the Cox proportional hazards model.* P* < 0.05 was considered statistically significant.

## Abbreviations

SCLC: small cell lung cancer
CDYL: chromodomain Y-like
CDKN1C: cyclin-dependent Kinase Inhibitor 1C
EZH2: enhancer of zeste homologue 2
H3K27me3: trimethylation of lysine 27 in histone 3
ChIP: chromatin immunoprecipitation
IHC: immunohistochemistry
CCK-8: cell counting kit-8 assay
BSP: bisulfite sequencing PCR
