# Supplementary material for: CDYL promotes the chemoresistance of small cell lung cancer by regulating H3K27 trimethylation at the CDKN1C promoter
Source: Theranostics. 2019 Jul 9;9(16):4717–29. doi: 10.7150/thno.33680 (PMC6643436; doi:10.7150/thno.33680)
Supplement: Supplementary file 1 — Supplementary figures and tables. [file thnov09p4717s1.pdf]

Supplementary Materials

Supplementary Figures

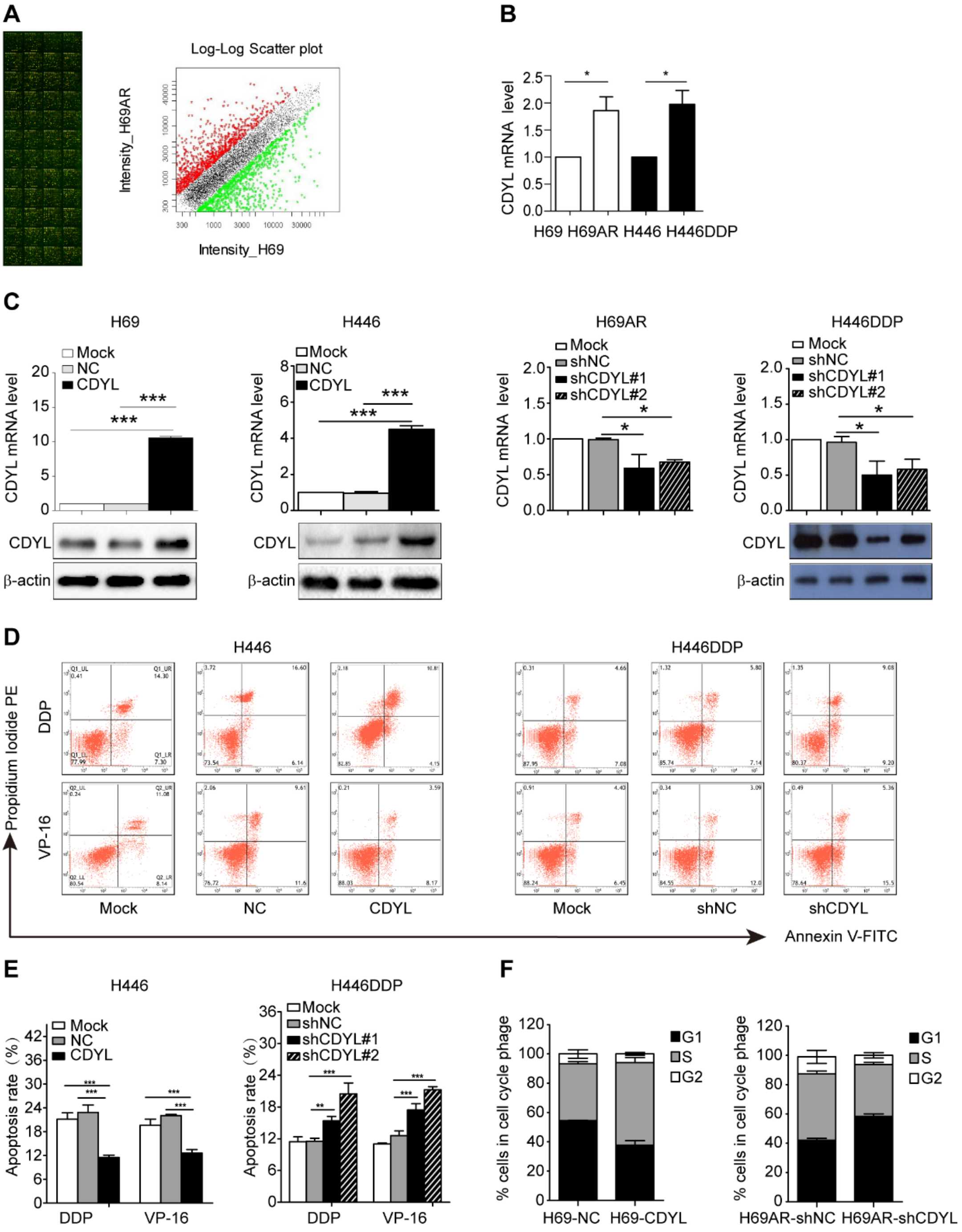

Fig S1. Effect of CDYL on chemoresistance in SCLC cells. (A) The cDNA expression profile revealed that CDYL is differentially expressed between H69AR cells and H69 cells. (B) RT-qPCR analysis of CDYL expression in sensitive and

resistant H69-H69AR and H446-H446DDP SCLC cells, respectively. (C) RT-qPCR and Western blot analyses of CDYL levels in SCLC cells transfected with the LV5-CDYL lentivirus, shRNAs targeting CDYL (shCDYL#1 and shCDYL#2), and the corresponding control vectors. (D) Representative dot plots of flow cytometry data showing the effect of CDYL on apoptosis in H446 and H446DDP cells in response to exposure to cytotoxic drugs. (E) Summary of the cumulative data showing the percentage of apoptotic cells in CDYL-overexpressing H446 cells (left panel), CDYL knockdown H446DDP cells (right panel) and the corresponding controls. (F) Cell cycle progression was determined in CDYL-overexpressing and CDYL knockdown SCLC cells after chemotherapeutic drug exposure using flow cytometry.

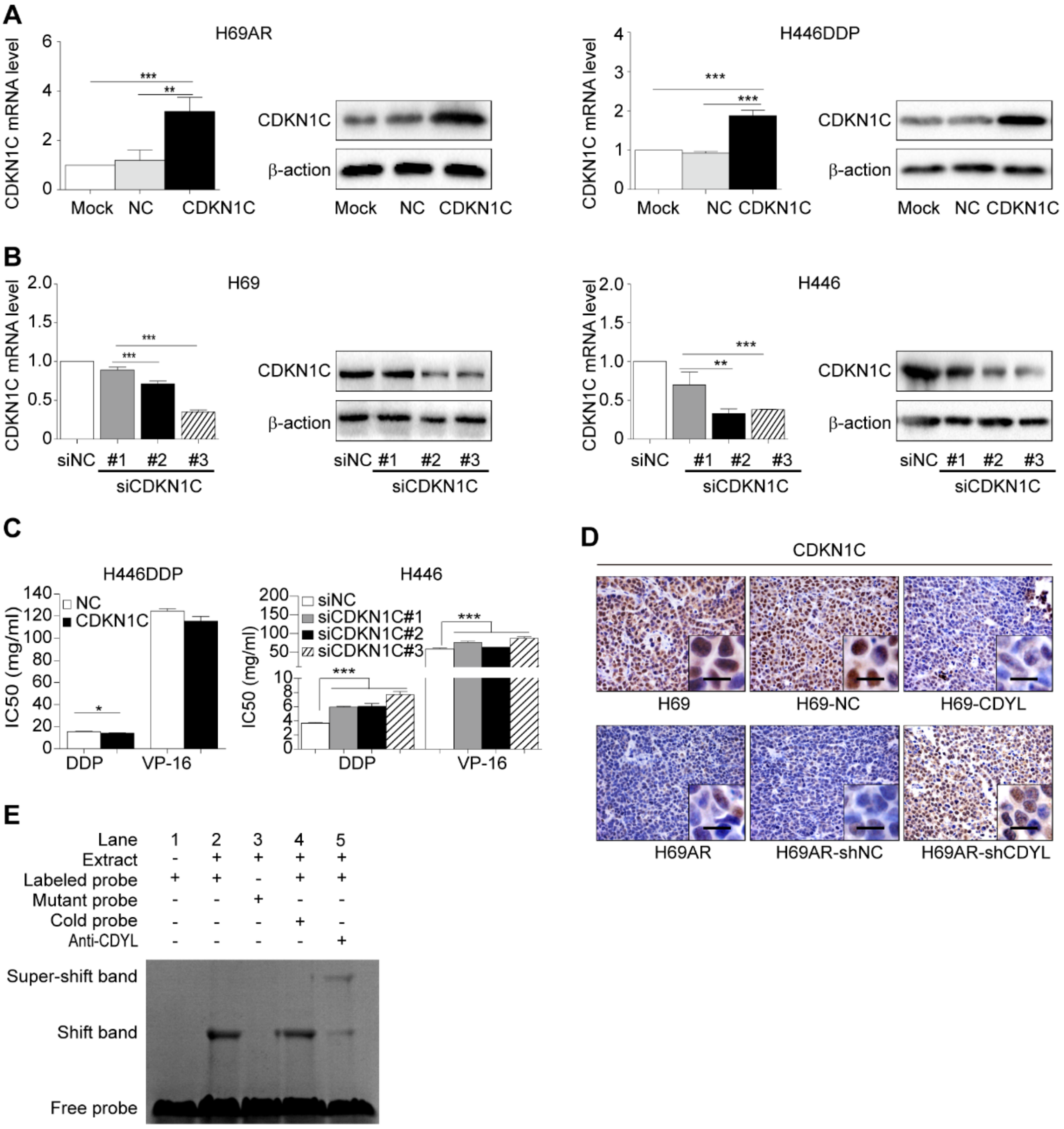

Fig S2. Effect of CDKN1C on chemoresistance in SCLC cells. (A-B) RT-qPCR and Western blot analyses of CDKN1C levels in cells transfected with pcDNA3.1-CDKN1C (A), siRNA-CDKN1C (B) and corresponding control vectors. (C) Comparison of IC50 values in H446DDP-CDKN1C (left panel) or H446-siCDKN1C (right panel) cells and corresponding controls following exposure to cytotoxic agents [(DDP: cisplatin, 5 μg/ml; VP-16: etoposide, 200 μg/ml) for 24 h]. (D) IHC staining for CDKN1C in xenograft tumours with different CDYL levels. Scale bars, 50 μm. (E) CDYL EMSA assessing the binding of the nuclear extract to

the CDKN1C promoter.

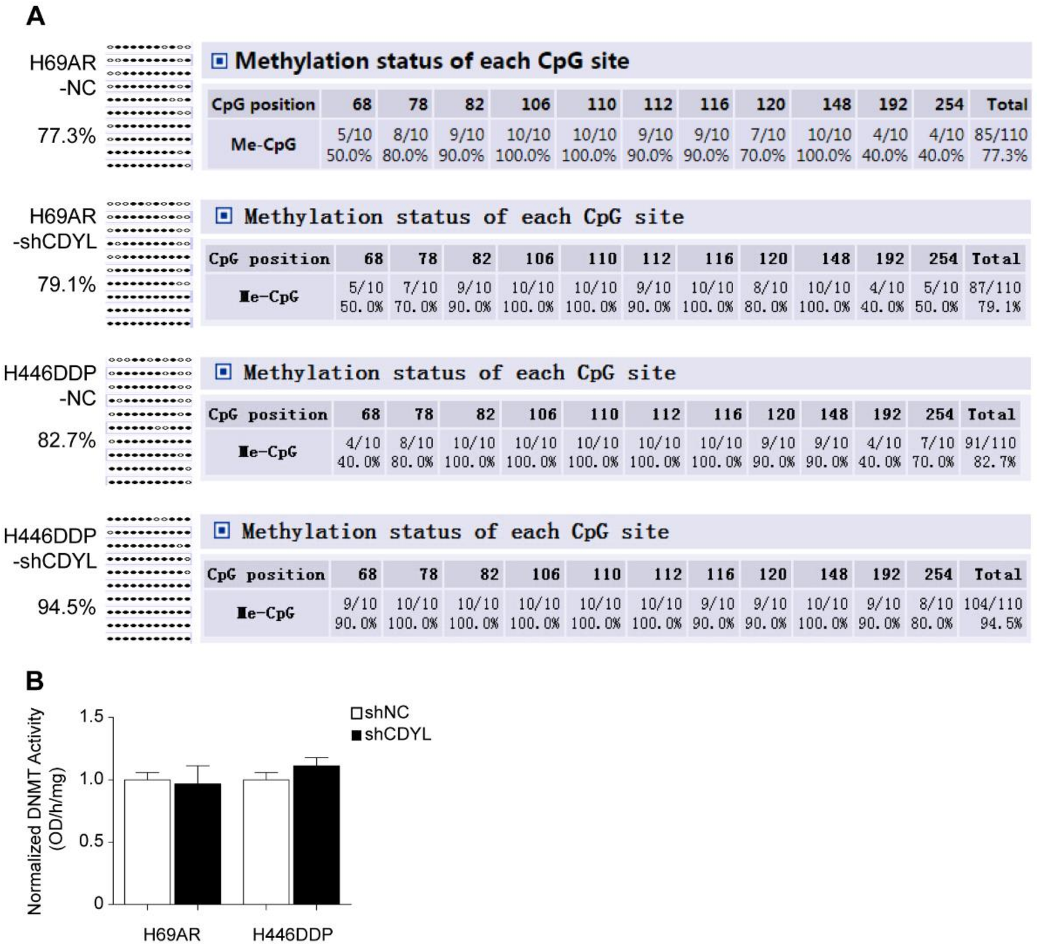

Fig S3. Level of DNA methylation at the CDKN1C promoter in SCLC. (A) BSP analysis of the level of DNA methylation at the CDKN1C promoter in CDYL-deficient cells and control cells. (B) The total DNMT activity was detected with nuclear extracts from CDYL-deficient cells and control cells; data were normalized to control cells, n = 3.

Supplementary Tables

Table S1. Sequences of shRNA

shRNA	sequence

shCDYL#1 (sh1376)	GUGGACUUGACUUUAUUUA
shCDYL#2 (sh1624)	GGCUGUUCUACCGUUAUGU

Table S2. Sequences of siRNA

**RNAi**	**Sense sequence (5’-3’)**	**Antisense sequence (5’-3’)**
CDKN1C-332	GGACCUUCCCAGUACUAGUTT	ACUAGUACUGGGAAGGUCCTT
CDKN1C-455	GCUGGGAUUACGACUUCCATT	UGGAAGUCGUAAUCCCAGCTT
CDKN1C-1091	UCUCCGAUUUCUUCGCCAATT	UUGGCGAAGAAAUCGGAGATT

Table S3. Primers for real-time PCR.

mRNA	Forward primer (5’-3’)	Reverse primer (5’-3’)
CDKN1A	GTCCAGCGACCTTCCTCATC	CCATAGCCTCTACTGCCACCA
CDKN1C	AAGAGTCCACCACCGGACAG	AGAGGAGAGGACAGCGAGAAGA
CDKN2A	GGAGAGGGGGAGAGCAGGCAG	TCCAGAGTCGCCCGCCATC
	GGATCCTTGCAGGTCATGATGTT	CTCGAGTTTGTTGACAAGTAACCC
CDKN2D	TGGAAGGAAGTG	AGTAAGGCT
BRMS1	CAGAGGCAGCTCCAGGTCTT	ATGGCTGTCCAGTCCTCCAG
NEUROD1	CACGAGGCAGACAAGAAGGA	CCTCCTCTTCCAGGTCCTCA
FOS	ACTCCAAGCGGAGACAGACC	TGAGCTGCCAGGATGAACTC
RhoA	AGTTGTCCTCCTGCCTCAGC	GGCTCCATCACCAACAATCA
EGR2	TTCCAGTGTCGGATCTGCAT	TCGGCCACAGTAGTCACAGG
cofilin	CTCTCGTCTTCTGCGGCTCT	ACGCACCTTCATGTCGTTGA
CD83	AAGTGAGGAAGCCAGGTCCA	CATCCATGCAACACTTCGTG
GPC2	TAAGGTGCCGGTGTCTGAAG	CTGAGACAGCCACGAACCAC
FAS	TTACGAGTGACTTGGCTGGAG	TTGAGCAATCCTCCGAAGTG
EGFR	AATGCGTGGACAAGTGCAAC	TGTGCAGGTGATGTTCATGG
STAT-3	CATCCTGGCTAACACGGTGA	AAGCGATTCTCCTGCCTCAG

ANXA1	GATGTCGCTGCCTTGCATAA	TCTGTTGACGCTGTGCATTG
MMP7	GACTCACCGTGCTGTGTGCT	TGAGATAGTCCTGAGCCTGTTCC
TWIST	GGCCAGGTACATCGACTTCC	CATCCTCCAGACCGAGAAGG
E-cadherin	TGAGGCCAAGCAGCAGTACA	GGCTTCATTCACATCCAGCA
MMP9	TCATCTTCCAAGGCCAATCC	GCAGAAGCCGAAGAGCTTGT
MMP2	CAAGATGGAGGTGCCTGGTT	AGCCAAGCGGTCTAAGTCCA
BNDF	TTCCACCAGGTGAGAAGAGT	ACTAATACTGTCACACACGC
TrkC	CCCCCATTTGCGTTATATAAACC	CACACGTGGGGGATAGTAGACA
SCN8A	CCAAACTAAAGGTGCACGCC	TGGAAGTCACCATTCCGGTG

Table S4. Primers for ChIP-qPCR target CDKN1C gene promoter

Antibodies	Forward (5'-3')	Reverse (5'-3')
CDYL	TGTAGTGCCTTTCCTCTACT	AAATCCTGCAAGCGCGGGGT
EZH2	CTGGGGGCAAGAATGACAGG	GCTAGTGCCCTAAATCCTGC
H3K37me3	TGCCTTTCCTCTACTGCTCT	GGGGGTGCTAGTCCCAAGGG

Table S5. Probe Sequences of EMSA

	sequence
Probe	GGGCCCGGAACTCACGACCCCCGACAGCGGTCTCCCCCGCCCCCC CAACCCCG
Mutant probe	GGGCCCGGAAACAGATGAAAAATGAGTATTCCTCCCCCGCCCCCC CAACCCCG.

Table S6. Primer sequences for BSP

Gene promoter	Forward primer (5’-3’)	Reverse primer (5’-3’)
CDKN1C	GTGGGTTAAAAGGGAGGTAGT	CCTTCTCAACCTTAAACCAAAC
